# Optimization of vision transformer-based detection of lung diseases from chest X-ray images

**DOI:** 10.1186/s12911-024-02591-3

**Published:** 2024-07-08

**Authors:** Jinsol Ko, Soyeon Park, Hyun Goo Woo

**Affiliations:** 1 Department of Physiology, Ajou University School of Medicine, Suwon, Republic of Korea; 2 Department of Biomedical Science, Graduate School, Ajou University, Suwon, Republic of Korea; 3 Ajou University School of Medicine, Suwon, Republic of Korea

**Keywords:** Optimizer, Vision transformer, Lung disease, X-ray

## Abstract

**Background:**

Recent advances in Vision Transformer (ViT)-based deep learning have significantly improved the accuracy of lung disease prediction from chest X-ray images. However, limited research exists on comparing the effectiveness of different optimizers for lung disease prediction within ViT models. This study aims to systematically evaluate and compare the performance of various optimization methods for ViT-based models in predicting lung diseases from chest X-ray images.

**Methods:**

This study utilized a chest X-ray image dataset comprising 19,003 images containing both normal cases and six lung diseases: COVID-19, Viral Pneumonia, Bacterial Pneumonia, Middle East Respiratory Syndrome (MERS), Severe Acute Respiratory Syndrome (SARS), and Tuberculosis. Each ViT model (ViT, FastViT, and CrossViT) was individually trained with each optimization method (Adam, AdamW, NAdam, RAdam, SGDW, and Momentum) to assess their performance in lung disease prediction.

**Results:**

When tested with ViT on the dataset with balanced-sample sized classes, RAdam demonstrated superior accuracy compared to other optimizers, achieving 95.87%. In the dataset with imbalanced sample size, FastViT with NAdam achieved the best performance with an accuracy of 97.63%.

**Conclusions:**

We provide comprehensive optimization strategies for developing ViT-based model architectures, which can enhance the performance of these models for lung disease prediction from chest X-ray images.

**Supplementary Information:**

The online version contains supplementary material available at 10.1186/s12911-024-02591-3.

## Background

Deep learning algorithms have demonstrated remarkable success in distinguishing lung diseases by analyzing chest X-rays. Convolutional neural networks (CNNs), in particular, have achieved promising results in this domain [1–4]. However, the introduction of Vision Transformers (ViTs) has opened up new avenues for lung disease classification [5]. Unlike CNNs, ViTs utilize self-attention mechanisms, converting images into sequences of image patches that are then processed by a transformer [6]. This novel approach has yielded state-of-the-art performance in various computer vision tasks, including image classification, object detection, and segmentation. Indeed, ViTs have surpassed CNNs in detecting COVID-19 from chest X-rays, achieving accuracies above 96% [7–10]. Moreover, new ViT-based models, such as FastViT and CrossViT, have also emerged and exhibited promising results. FastViT is a cutting-edge hybrid form of ViT that strikes an optimal balance between latency and accuracy [11]. CrossViT employs a dual-branch transformer with a cross-attention mechanism, generating stronger image features [12].

On the other hand, enhancing the accuracy of deep learning models remains a crucial area of research. Data augmentation has proven to be an effective technique for improving neural network precision. However, not all models benefit equally from augmentation, necessitating the exploration of broadly applicable methods such as loss function optimization [13]. Indeed, various optimization techniques have demonstrated varying performances in training CNN models. For instance, the Xception model, which was pretrained for classifying chest X-rays into normal, COVID-19, and pneumonia categories, achieved the highest accuracy with the Root Mean Square Propagation (RMSProp) optimizer [14]. Meanwhile, other studies have reported superior performance with the Adaptive Moment Estimation (Adam) optimizer [15, 16] or Adaptive Gradient (AdaGrad) optimizer [17] when coupled with CNN models. These findings underscore the model-dependent nature of optimizer performance. Similarly, ViT models exhibit varying performance depending on the chosen optimizer. Rectified Adam (RAdam) [8, 9], Adam with cosine decay [7], NovoGrad [10], and AdaBelief [18] have been applied to ViTs. Notwithstanding, a comprehensive comparison of optimization methods for ViT models in chest X-ray image classification has not been conducted.

This study aimed to identify the best performing optimization method in training ViT-based models for predicting lung diseases from chest X-ray images. We evaluated the following six optimization methods known to produce promising results in computer vision: Adam, Adam with weight decay (AdamW), Nesterov accelerated Adam (NAdam), RAdam, Stochastic Gradient Descent with weight decay (SGDW), and Momentum. Stochastic Gradient Descent (SGD), a cornerstone optimizer in modern neural networks, paved the way for achieving minimal loss values [19, 20]. Enhancements to SGD, such as Momentum and Nesterov acceleration techniques, have improved its effectiveness, resulting in faster convergence and higher accuracy with fewer steps [13]. In addition, several advanced algorithms, such as AdaGrad, RMSProp, Adadelta, and Adam, have been proposed to effectively navigate complex functions with local extremes. The Adam-based algorithms utilize exponential moving averages and excel at refining minimization processes in functions with multiple extremes. A notable advantage of these methodologies lies in their ability to derive corrected estimates for bias, effectively countering the effects of initial bias settings [13]. Moreover, building upon the success of the Adam optimizer, several variants, including AdamW, NAdam, and RAdam, have emerged as promising alternatives. These variants introduce subtle refinements to the original algorithm, incorporating features such as weight decay or adaptive learning rate adjustments to further enhance its performance and robustness. In this study, we conducted a comprehensive comparison of these optimization algorithms, including Adam and its variants (AdamW, NAdam, and RAdam) and the traditional Momentum and SGDW (a variant of SGD). This in-depth evaluation could address the best fitted optimizer according to the model and dataset.

## Methods

### Dataset

We obtained a publicly available chest X-ray dataset from an open-source repository, details of which can be found in the availability of data and materials section [21–31]. The dataset comprised 19,003 images, categorized into six disease classes: COVID-19 (*n* = 3,616), Viral Pneumonia (*n* = 1,345), Bacterial Pneumonia (*n* = 2,772), Middle East Respiratory Syndrome (MERS, *n* = 144), Severe Acute Respiratory Syndrome (SARS, *n* = 134), and Tuberculosis (*n* = 800). Additionally, the dataset included normal chest X-rays (*n* = 10,192) for reference.

### Model structure

The RGB values of the image data were normalized to an average of 0.5 and a standard deviation of 0.5. The image size was set to 224 x 224 pixels, and each image was divided into 16 x16 patches, which were then flattened and linearly projected to create patch embeddings. These embeddings were combined with position embeddings to preserve positional information. We utilized the ViT-B/16 model [5], which was pretrained on ImageNet data [32]. For each block, layer normalization and residual connection were applied [5]. Following recommendations from previous research [33], we trained the model with a batch size of 32 for 15 epochs, shuffling the dataset before each epoch.

We applied six different optimizers, including Adam, AdamW, NAdam, RAdam, SGDW, and Momentum (Supplementary Table 1). Each optimizer was tested on three different learning rates: 10^–4^, 10^–5^, and 10^–6^. All the parameters used in the models are summarized in Supplementary Table 2.

### Evaluation

The performance of each model was evaluated using four key metrics: accuracy, F1-score, precision, and recall [34]. Accuracy, precision, and recall are parameters that can effectively assess the performance of a model, and F1-score is known to be robust against data with imbalanced sample size [34, 35].

$$\text{Accuracy }= (\text{True positive }+\text{ True negative}) / (\text{True positive }+\text{ True negative }+\text{ False positive }+\text{ False negative})$$

$$\text{F}1-\text{score }=\text{ Harmonic mean of precision and recall }= 2\text{ x Precision x Recall }/ (\text{Precision }+\text{ Recall})$$

$$\text{Precision }=\text{ Positive Predictive Value }=\text{ True positive }/ (\text{True positive }+\text{ False positive})$$

$$\text{Recall }=\text{ Sensitivity }=\text{ True positive }/ (\text{True positive }+\text{ False negative})$$

## Results

### Classification of the overall classes with various models and optimizers

**Fig. 1:**
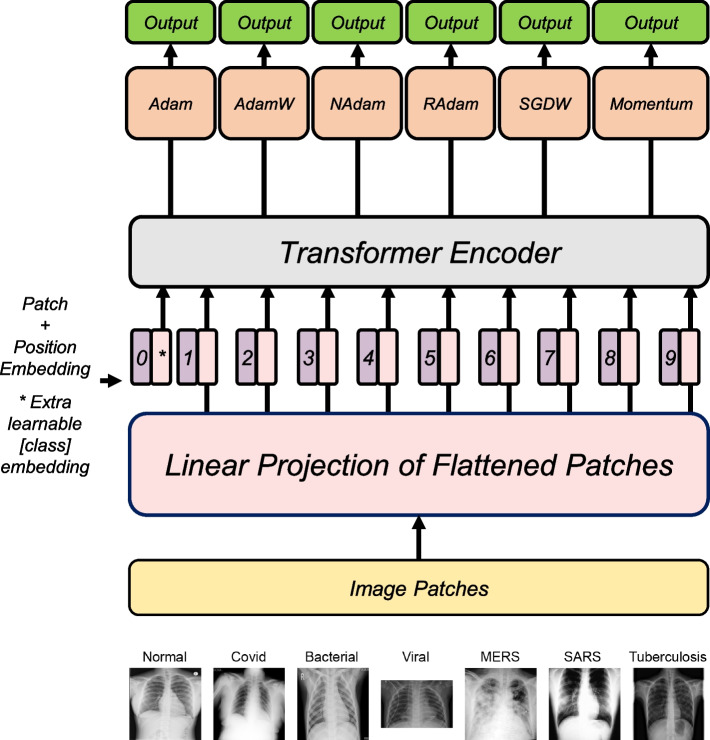
Schematic overview of the analysis workflow

The sample sizes for each class in the chest X-ray dataset ranged from 134 to 10,192 images. The presence of imbalanced sample sizes may compromise the learning process, leading to biased outcomes [36, 37]. Thus, we evaluated the effect of the imbalanced sample size by performing analysis using the 4 class dataset with sample sizes greater than 1,000 (i.e., Normal, COVID-19, Viral Pneumonia, and Bacterial Pneumonia). We compared these results with those from the complete dataset also including the small sample-sized classes (i.e., MERS, SARS, and Tuberculosis). The dataset was randomly split into the training dataset (80%) and test dataset (20%), and the model performance was evaluated with different learning rates of 10^–4^, 10^–5^, and 10^–6^. The overall structure of the model is demonstrated in Fig. 1.

**Fig. 2:**
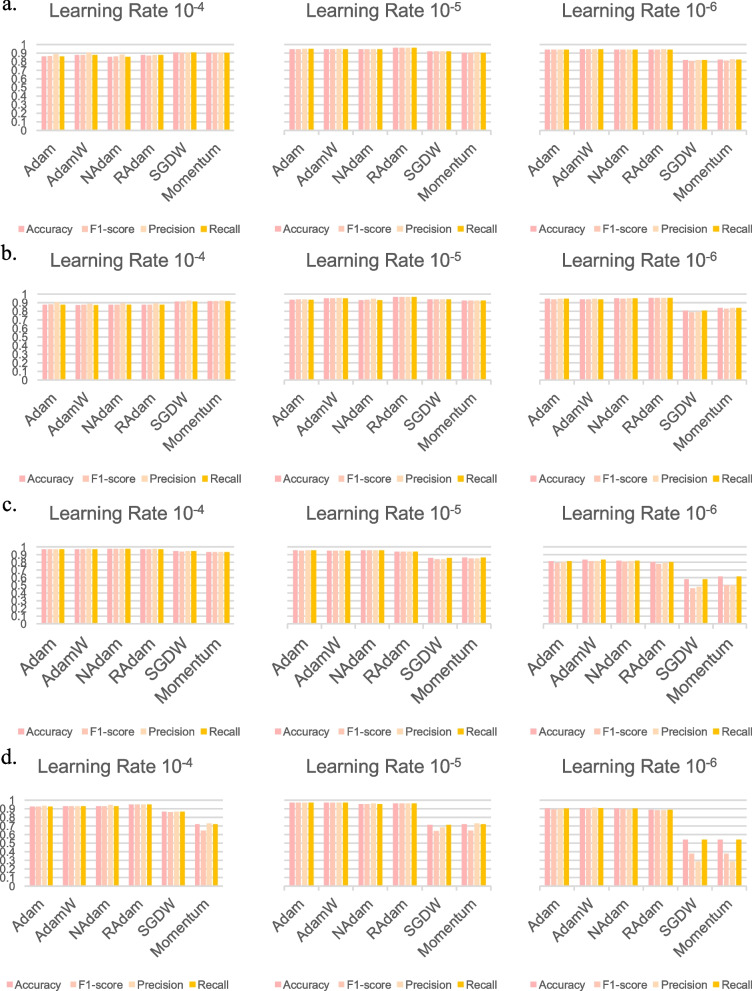
Classification of the overall classes with various models and optimizers. **A**, **B** The 4 class (**A**) and 7 class (**B**) datasets were classified using the ViT model with various optimizers (Adam, AdamW, NAdam, RAdam, SGDW, and Momentum), respectively. **C**, **D** The 7 class dataset was classified using the FastViT (**C**) or CrossViT (**D**) models with various optimizers, respectively. The evaluation metrics included accuracy, F1-score, precision, and recall, calculated at various learning rates of 10^–4^, 10^–5^, and 10^–6^

First, we evaluated the performance of the models with no optimizer, which revealed poor accuracies in each test of the ViT with 4 class (30.90%) and the ViT with 7 class (14.89%), respectively (Supplementary Fig. 1). FastViT (6.10%) and CrossViT (22.72%) with 7 class also showed poor performance, indicating the need for the use of an optimizer (Supplementary Fig. 2). Next, we evaluated the performance of the ViT model with different optimizers and learning rates (i.e., 10^–4^, 10^–5^, and 10^–6^) using the 4 class dataset. In general, ViT exhibited robust classification independent of the utilization of different optimizers (Fig. 2a, Supplementary Table 3, and Supplementary Fig. 3). However, SGDW and Momentum exhibited relatively lower accuracies (SGDW: 81.71%, Momentum: 82.18%) at learning rate 10^–6^ compared to the other optimizers (> 86%). Remarkably, RAdam showed the highest accuracy (95.87%, learning rate 10^–5^), while Adam achieved the highest F1-score (94.71%, learning rate 10^–5^). Adam-based optimizers (Adam, AdamW, NAdam, and RAdam) consistently demonstrated superior performance across all metrics, outperforming other optimizers (SGDW and Momentum). This finding could be attributed to the adaptive momentum algorithm employed by Adam-based optimizers. In addition, we evaluated the performance of ViT with the imbalanced sample-sized dataset (i.e., 7 class dataset). RAdam at learning rate 10^–5^ demonstrated the highest accuracy (96.61%) and F1-score (96.62%) in the 7 class dataset (Fig. 2b, Supplementary Table 3, and Supplementary Fig. 4).

In addition to ViT, when we evaluated the performance of FastViT and CrossViT, FastViT showed best performance with NAdam (accuracy 97.63%, F1-score 97.64%, learning rate 10^–4^; Fig. 2c, Supplementary Table 3, and Supplementary Fig. 5), while CrossViT showed best performance with AdamW (accuracy 96.95%, F1-score 96.94%, learning rate 10^–5^; Fig. 2d, Supplementary Table 3, and Supplementary Fig. 6). These results indicate that Adam-based optimizers perform well in ViT-based models. Comparing results of the imbalanced 7 class dataset in all three models (ViT, FastViT, and CrossViT), both the highest accuracy and F1-score were achieved by FastViT with NAdam. This finding may indicate the robustness of NAdam against sample imbalance.

### The disease class prediction with various ViT models and optimizers

**Fig. 3:**
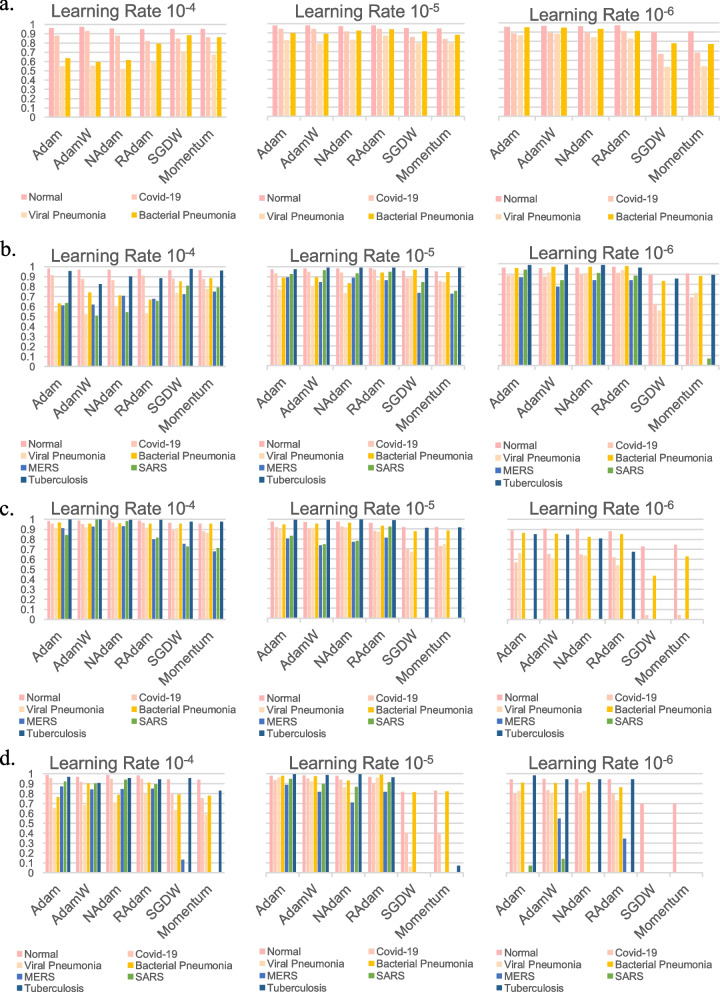
Classification of each disease class with various models and optimizers. **A**, **B** Each class in the 4 class (**A**) and 7 class (**B**) datasets was classified using the ViT model with various optimizers (Adam, AdamW, NAdam, RAdam, SGDW, and Momentum), respectively. **C**, **D** Each class in the 7 class dataset was classified using the FastViT (**C**) or CrossViT (**D**) models with various optimizers, respectively. The evaluation metrics included accuracy, F1-score, precision, and recall, calculated at various learning rates of 10^–4^, 10^–5^, and 10^–6^

Next, we sought to evaluate whether the prediction performance for each disease class varied depending on the models and optimizers by calculating F1-scores. In the 4 class classification by ViT, the Normal class exhibited robust prediction performance across all optimizers, showing the highest F1-score with RAdam (98.05%, learning rate 10^–5^), whereas the Viral Pneumonia class with NAdam showed the lowest performance (52.45%, learning rate 10^–4^) (Fig. 3a and Supplementary Table 4). In addition, we also evaluated the performance of ViT with varying optimizers in the 7 class classification. Similarly, RAdam showed satisfactory performance for predicting the Normal class (98.73%, learning rate 10^–5^), while AdamW was the best optimizer for the class of Tuberculosis (99.07%, learning rate 10^–6^) (Fig. 3b and Supplementary Table 4). In FastViT and CrossViT, Tuberculosis was the best predicted class (FastViT: Adam, 100%, learning rate 10^–4^; CrossViT: NAdam, 99.69%, learning rate 10^–5^) in both models (Fig. 3c, d and Supplementary Table 4).

## Discussion

In this study, we evaluated the performance of different optimizers in ViT-based prediction of lung diseases from chest X-ray images. By comparing the performance of six different optimizers, we found that Adam-based optimizers displayed superior performance compared to SGDW and Momentum. This might be because Adam-based optimizers are implemented with an adaptive momentum, adapting learning rates to the parameters. Similarly, a previous study has demonstrated that SGD and Momentum increased error rates with the learning rate lower than 10^–5^ in CNN-based classification of brain MRI [38]. These results consistently suggest that Adam-based optimizers are well suited for ViT-based models in predicting lung diseases using chest X-rays.

We demonstrated that RAdam showed optimal performance in predicting the balanced 4 class dataset in the ViT model. Supporting this, RAdam has shown satisfactory performance in ViT-based COVID-19 classification, although Adam or RMSProp did not [8, 9]. RAdam is a recent technique that combines the strengths of both Adam and SGD, ensuring swift convergence without easily succumbing to local optima. RAdam rectifies the variance of the adaptive learning rate term, which can make the variance consistent. Therefore, the convergence of RAdam is largely unaffected by the initial learning rate value [39].

In the imbalanced 7 class dataset, NAdam in FastViT had the highest accuracy (97.63%), which is comparable to previous ViT models for COVID-19 detection [8, 40–42]. NAdam modifies the momentum technique applied in Adam to the Nesterov accelerated gradient (NAG). By combining the advantages of Adam and NAG, NAdam can find the global minimum faster and more accurately than Adam. Unlike Adam, NAdam is based on NAG, which does not calculate the gradient value at the current position but after moving in the direction of the momentum. Thus, it computes the gradient at a new position [43].

For each class prediction, Normal and Tuberculosis were classified better than other classes. This might be due to the ambiguous feature of X-ray images among COVID-19, Viral Pneumonia, Bacterial Pneumonia, MERS, and SARS classes. The limitation of this study is that the models struggled with detecting small sized classes such as MERS and SARS. Further studies with diverse cases and varied sample sizes and diseases are needed for better performance. Additionally, we only focused on transformer models such as ViT, FastViT, and CrossViT. Future studies may deepen this field of study by investigating other optimizers in various computer vision models, such as hybrid models.

## Conclusions

In summary, our analyses of ViT-based models with various optimizers showed varying performance in predicting lung diseases from the chest X-ray images. Adam-based optimizers showed better performance in predicting disease classes. In the balanced dataset, RAdam was the best performing optimizer, while NAdam with FastViT showed the best accuracy in the imbalanced dataset. For the prediction of each disease class, Normal and Tuberculosis were well predicted compared to other classes. Our results might help develop the optimized algorithms with different model architectures and optimizers.

### Supplementary Information

Supplementary Material 1.

Supplementary Material 2.

## Data Availability

The datasets generated and analyzed during the current study are available in the BIMCV, GitHub, SIRM, eurorad, figshare, and Kaggle repository, [1] https://bimcv.cipf.es/bimcv-projects/bimcv-covid19/#1590858128006-9e640421-6711 [2] https://github.com/ml-workgroup/covid-19-image-repository/tree/master/png [3] https://sirm.org/category/senza-categoria/covid-19/ [4] https://eurorad.org [5] https://github.com/ieee8023/covid-chestxray-dataset [6] https://figshare.com/articles/COVID-19_Chest_X-Ray_Image_Repository/12580328 [7] https://github.com/armiro/COVID-CXNet [8] https://www.kaggle.com/c/rsna-pneumonia-detection-challenge/data [9] https://www.kaggle.com/paultimothymooney/chest-xray-pneumonia [10] https://www.kaggle.com/datasets/057e1b6dc41d9691e59dded4445fa8cc2f0b4b5cbcb49aef9583d95233799d5a [11] https://www.kaggle.com/datasets/tawsifurrahman/tuberculosis-tb-chest-xray-dataset

## Abbreviations

CNN: Convolutional neural network
ViT: Vision transformer
COVID-19: Coronavirus disease 2019
RMSProp: Root mean square propagation
Adam: Adaptive moment estimation
AdaGrad: Adaptive gradient
RAdam: Rectified Adam
AdamW: Adam with weight decay
NAdam: Nesterov accelerated Adam
SGDW: Stochastic gradient descent with weight decay
SGD: Stochastic gradient descent
MERS: Middle east respiratory syndrome
SARS: Severe acute respiratory syndrome
