# Supplementary material for: Optimization of vision transformer-based detection of lung diseases from chest X-ray images
Source: BMC Med Inform Decis Mak. 2024 Jul 8;24:191. doi: 10.1186/s12911-024-02591-3 (PMC11232177; doi:10.1186/s12911-024-02591-3)

Supplementary material

Supplementary Figures

Supplementary Figure 1. Performance estimation of overall and each class prediction without optimizer.

Supplementary Figure 2. Confusion matrix for 4 Class Dataset of ViT, 7 Class Dataset of ViT, 7 Class Dataset of FastViT, and 7 Class Dataset of CrossViT using no optimizer

Supplementary Figure 3. Confusion matrix for 4 Class Dataset of ViT

Supplementary Figure 4. Confusion matrix for 7 Class Dataset of ViT

Supplementary Figure 5. Confusion matrix for 7 Class Dataset of FastViT

Supplementary Figure 6. Confusion matrix for 7 Class Dataset of CrossViT

Supplementary Figure 1. Performance estimation of overall and each class prediction with out optimizer

(A) Barplots show all of the evaluation (accuracy, F1-score, precision, and recall) for predicting 4 Class Dataset of ViT, 7 Class Dataset of ViT, 7 Class Dataset of FastViT, and 7 Class Dataset of CrossViT using no optimizer.

(B) Barplots show the F1-scores for predicting each class in the datasets of 4 Class Dataset of ViT, 7 Class Dataset of ViT, 7 Class Dataset of FastViT, and 7 Class Dataset of CrossViT using no optimizer.

a.

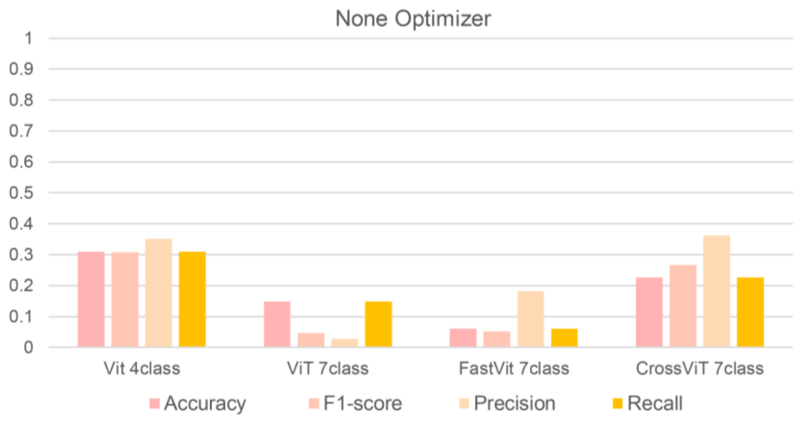

b.

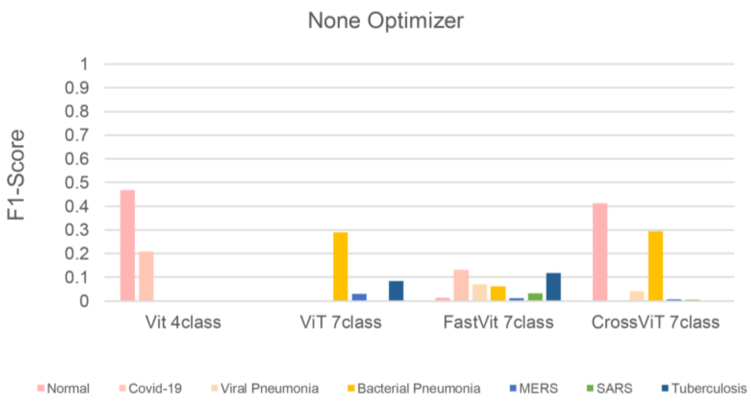

Supplementary Figure 2. Confusion matrix for 4 Class Dataset of ViT, 7 Class Dataset of ViT, 7 Class Dataset of FastViT, and 7 Class Dataset of CrossViT without optimizer

The confusion matrices show the models' prediction in (A) 4 Class Dataset of ViT, (B) 7 Class Dataset of ViT, (C) 7 Class Dataset of FastViT, and (D) 7 Class Dataset of CrossViT without optimizer.

a.

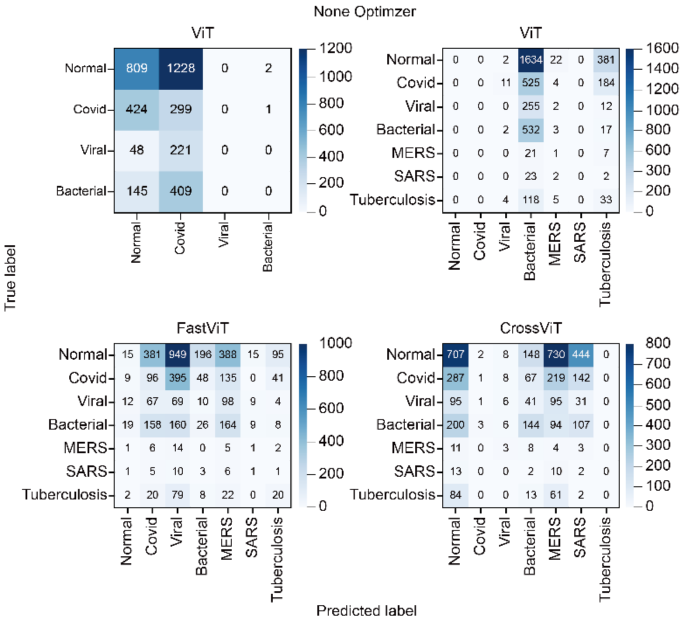

Supplementary Figure 3. Confusion matrix for 4 Class Dataset of ViT

The confusion matrices show the ViT models' prediction in 4 Class Dataset using different optimizers of Adam, AdamW, NAdam, RAdam, SGDW, and Momentum, respectively, with varying learning rates of 10^{-4} 10^{-5}, and 10^{-6}, respectively.

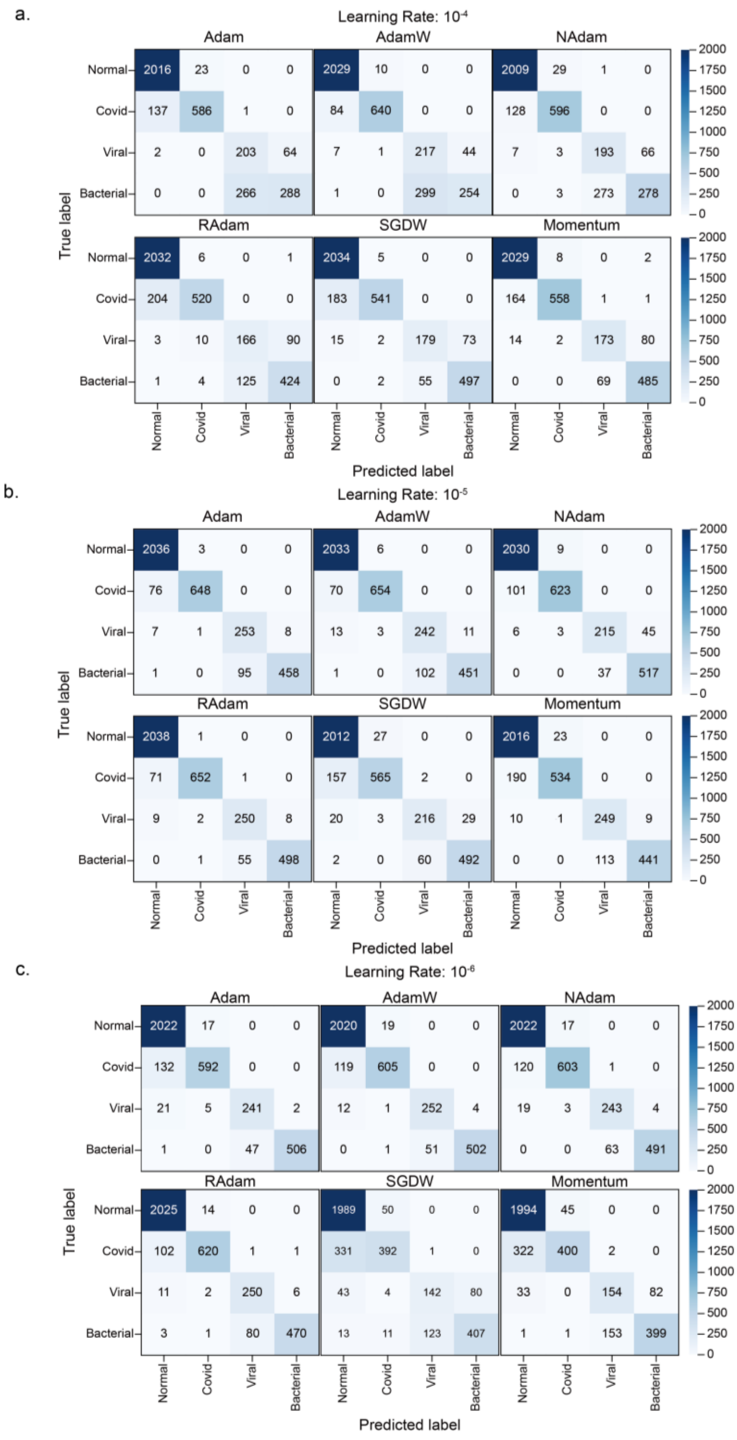

Supplementary Figure 4. Confusion matrix for 7 Class Dataset of ViT

The confusion matrices show the ViT models' prediction in 7 Class Dataset using different optimizers of Adam, AdamW, NAdam, RAdam, SGDW, and Momentum, respectively, with varying learning rates of 10^{-4} 10^{-5}, and 10^{-6}, respectively.

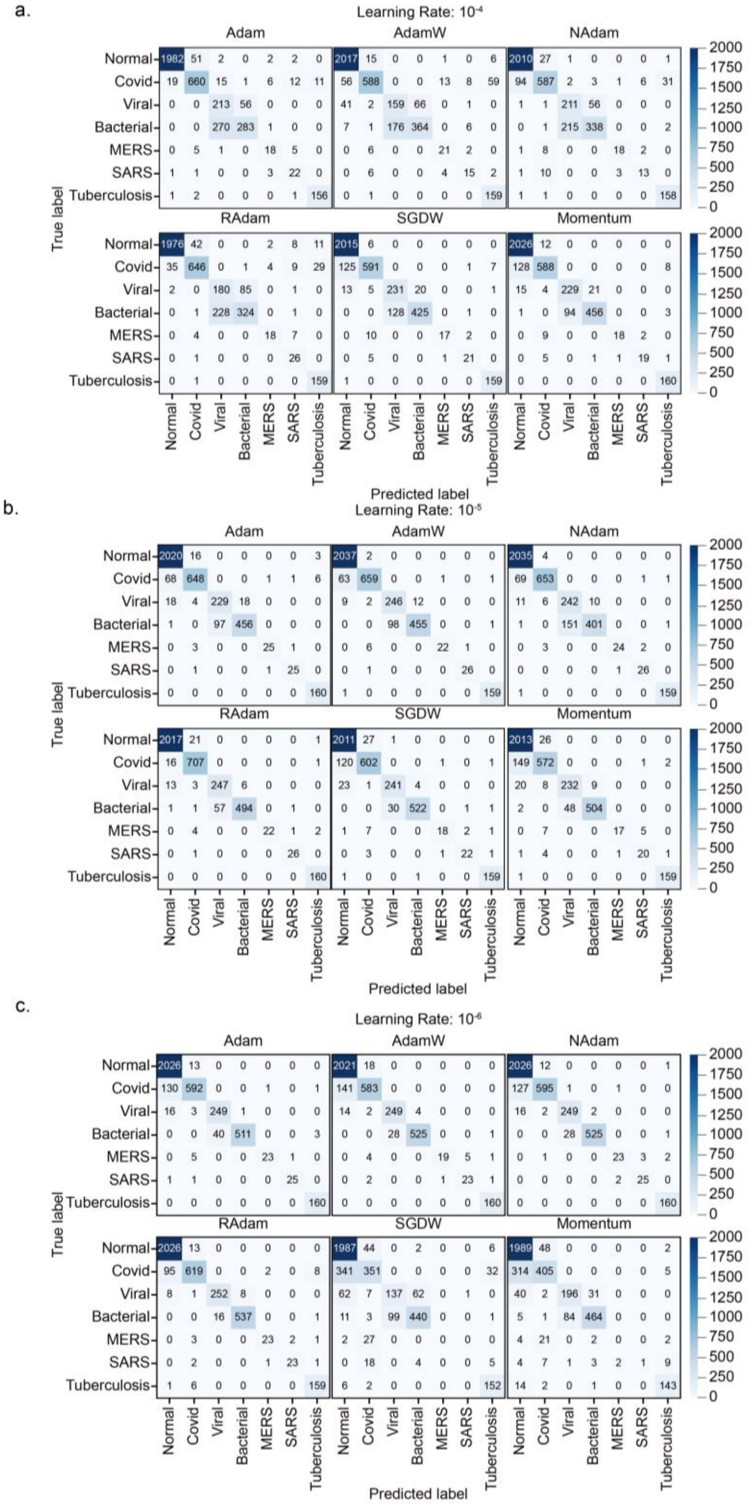

Supplementary Figure 5. Confusion matrix for 7 Class Dataset of FastViT

The confusion matrices show the FastViT models' prediction in 7 Class Dataset using different optimizers of Adam, AdamW, NAdam, RAdam, SGDW, and Momentum, respectively, with varying learning rates of 10^{-4} 10^{-5}, and 10^{-6}, respectively.

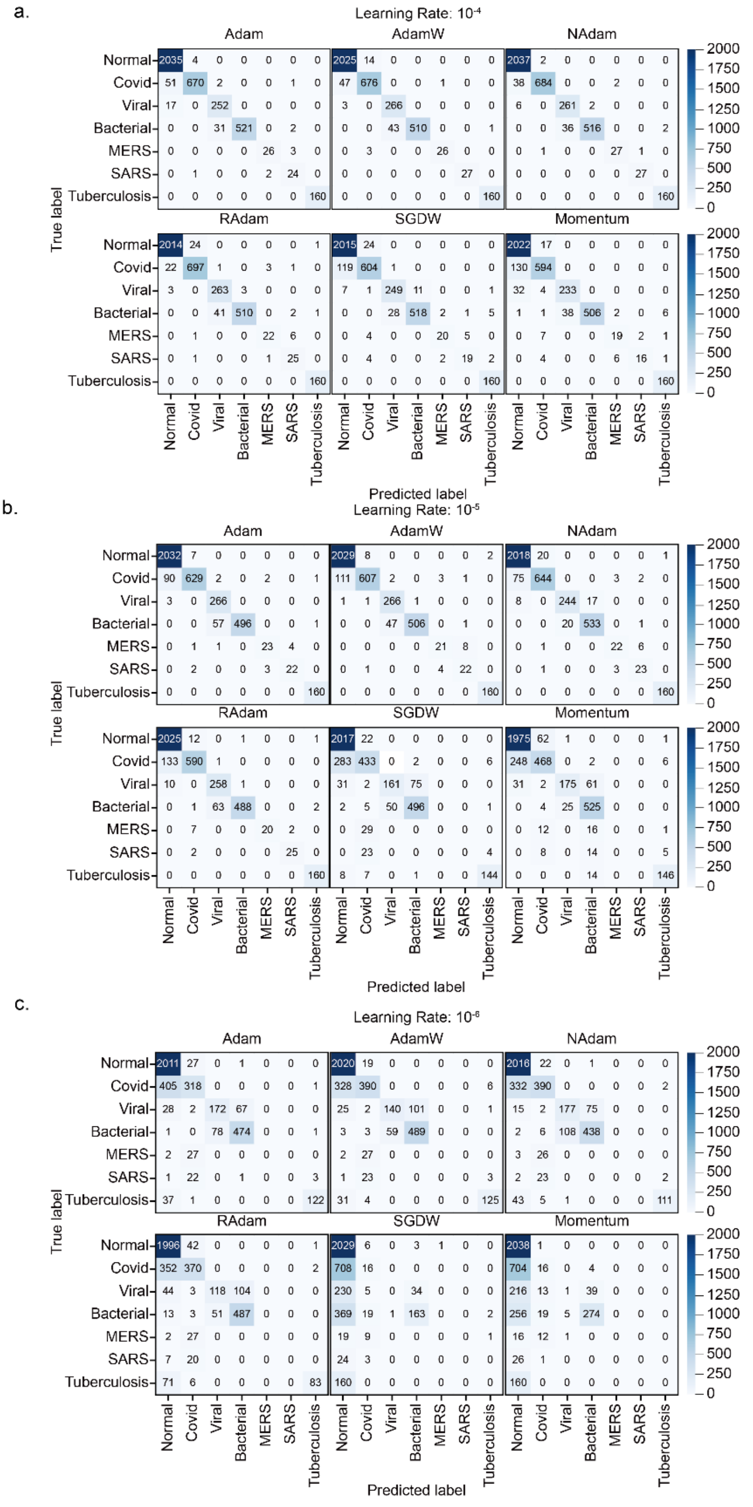

Supplementary Figure 6. Confusion matrix for 7 Class Dataset of CrossViT

The confusion matrices show the CrossViT models' prediction in 7 Class Dataset using different optimizers of Adam, AdamW, NAdam, RAdam, SGDW, and Momentum, respectively, with varying learning rates of 10^{-4} 10^{-5}, and 10^{-6}, respectively.

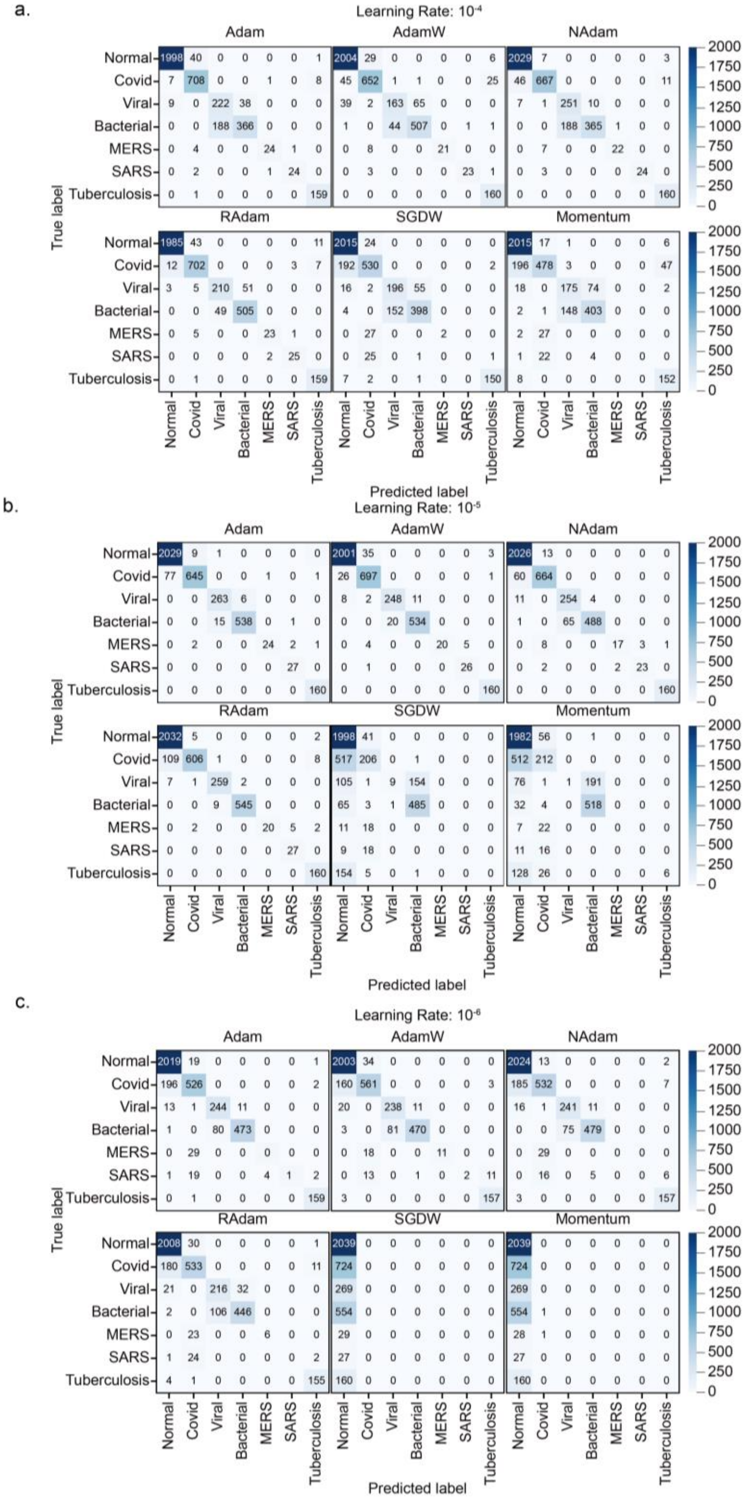

Supplement: Supplementary file 1 — Supplementary Material 1. [file 12911_2024_2591_MOESM1_ESM.pdf]
