# Supplementary material for: Optimization of vision transformer-based detection of lung diseases from chest X-ray images
Source: BMC Med Inform Decis Mak. 2024 Jul 8;24:191. doi: 10.1186/s12911-024-02591-3 (PMC11232177; doi:10.1186/s12911-024-02591-3)
Supplement: Supplementary file 2 — Supplementary Material 2. [file 12911_2024_2591_MOESM2_ESM.pdf]

Supplementary Table

Supplementary Table 1. List of optimizer

Supplementary Table 2. Parameters used for training the model

Supplementary Table 3. Estimation of the model performance by calculating all of the evaluation (accuracy, F1-score, precision, and recall)

Supplementary Table 4. Estimation of the model performance by calculating F1-scores of each class

Supplementary Table 1. List of optimizer

In this research, Adam, AdamW, NAdam, Radam, SGDW and Momentum were compared. Adam-based optimizers include Adam, AdamW, Nadam, and Radam, and non-Adam-based optimizers include SGDW and Momentum.

Optimizer	Description	Reference
Adam	Adam possesses features of both momentum (Qian 1999) and adaptive learning rate. Like momentum, exponentially decaying average of past gradients are accumulated, and like RMSprop, exponentially decaying average of past squared gradients are accumulated (Ruder 2016).	(Kingma and Ba 2014)
AdamW	AdamW is equivalent to Adam with decoupled weight decay. This optimizer not only provides weight decay effect by L2 regulation but also adds weight decay term to the weight update equation.	(Loshchilov and Hutter 2017)
NAdam	This optimizer is a combination of Adam and Nesterov accelerated gradient (NAG) (Nesterov 1983). In this optimizer, the look-ahead momentum vector is utilized. Unlike NAG, this optimizer first moves by the momentum value and then finds the gradient	(Dozat 2016)
RAdam	RAdam is a variant of Adam which incorporates rectification of the variance of the adaptive learning rate.	(Liu, Jiang et al. 2019)
SGDW	Addition of a direct weight loss term to the weight update expression as well as a weight loss effect on L2 regulation in SGD. Like AdamW, this optimizer not only provides weight decay effect by L2 reulation but also adds weight decay term to the weight update equation.	(Loshchilov, I., & Hutter, F. 2017)
Momentum	This optimizer adds momentum to SGD, which allows the model to converge faster. It remembers and takes into account the past movement direction when it moves.	(Qian 1999)

Supplementary Table 2. Parameters used for training the model

During pre-processing, images were resized to 224 x 224 and for normalization, mean and standard deviation were 0.5. Batch size was 32 for training, 1 for test.

**Parameters**	**Pre-processing**	Resize	224 x 224
		Normalize	Mean: 0.5
			Standard deviation: 0.5
	**Batch size**	Train	32
		Test	1
	**Epoch size**		15
	**Adam**	Betas	0.9, 0.999
		Eps	10^{-8}
		Weight decay	10^{-6}
	**AdamW**	Betas	0.9, 0.999
		Eps	10^{-8}
		Weight decay	10^{-6}
	**NAdam**	Betas	0.9, 0.999
		Eps	10^{-8}
		Weight decay	10^{-6}
	**Radam**	Betas	0.9, 0.999
		Eps	10^{-8}
		Weight decay	10^{-6}
	**SGDW**	Momentum	0.9
		Weight decay	10^{-6}

	**Momentum**	momentum	0.9
		Weight decay	0

Supplementary Table 3. Estimation of the model performance by calculating all of the evaluation (accuracy, F1-score, precision, and recall)

The table shows the accuracy, F1-score, precision, and recall of 4 Class Dataset of ViT, 7 Class Dataset of ViT, 7 Class Dataset of FastViT, and 7 Class Dataset of CrossViT using different optimizers of Adam, AdamW, NAdam, RAdam, SGDW, and Momentum, respectively. The models are tested with each learning rate 10^{-4}, 10^{-5}, and 10^{-6}.

**ViT 4 class (Normal, Covid, Viral Pneumonia, Bacterial Pneumonia)**							
	**Learning Rate**	**Accuracy**	**F1-score**	**Precision**	**Recall**		
**Adam**	10^{-4}	0.862521	0.863579	0.884996	0.862521		
	10^{-5}	0.946737	0.947120	0.953102	0.946737		
	10^{-6}	0.937256	0.936320	0.939675	0.937256		
**AdamW**	10^{-4}	0.875627	0.876192	0.905647	0.875627		
	10^{-5}	0.942554	0.943067	0.948777	0.942554		
	10^{-6}	0.942276	0.941628	0.944751	0.942276		
**NAdam**	10^{-4}	0.876185	0.873172	0.880203	0.876185		
	10^{-5}	0.943949	0.942964	0.944331	0.943949		
	10^{-6}	0.936698	0.936158	0.940059	0.936698		
**RAdam**	10^{-4}	0.876185	0.873172	0.880203	0.876185		
	10^{-5}	0.958728	0.933658	0.961121	0.958728		
	10^{-6}	0.938371	0.938228	0.943181	0.938371		
**SGDW**	10^{-4}	0.906581	0.903055	0.908839	0.906581		
	10^{-5}	0.916062	0.914400	0.917716	0.916062		
	10^{-6}	0.817066	0.806970	0.818324	0.817066		
**Momentum**	10^{-4}	0.904908	0.902071	0.906402	0.904908		
	10^{-5}	0.903514	0.901915	0.913493	0.903514		
	10^{-6}	0.821807	0.813785	0.829074	0.821807		
**None**	-	0.308979	0.307418	0.350565	0.308979		
**ViT 7 class (Normal, Covid-19, Viral Pneumonia, Bacterial Pneumonia, MERS, SARS, Tuberculosis)**							
	**Learning Rate**	**Accuracy**	**F1-score**	**Precision**	**Recall**		
**Adam**	10^{-4}	0.876907	0.880873	0.904446	0.876907		
	10^{-5}	0.937138	0.937420	0.941137	0.937138		
	10^{-6}	0.943188	0.942159	0.945203	0.943188		
**AdamW**	10^{-4}	0.874014	0.874846	0.884861	0.874014		
	10^{-5}	0.947922	0.948287	0.953301	0.947922		
	10^{-6}	0.941610	0.940206	0.942991	0.941610		
**NAdam**	10^{-4}	0.877170	0.877955	0.891885	0.877170		
	10^{-5}	0.931089	0.932010	0.943217	0.931089		
	10^{-6}	0.947659	0.946633	0.949359	0.947659		
**RAdam**	10^{-4}	0.875592	0.879046	0.892418	0.875592		
	10^{-5}	0.966070	0.966216	0.967841	0.966070		
	10^{-6}	0.957128	0.956352	0.957609	0.957128		
**SGDW**	10^{-4}	0.914519	0.914012	0.922545	0.914519		
	10^{-5}	0.940295	0.939205	0.940806	0.940295		
	10^{-6}	0.806681	0.787885	0.790141	0.806681		
**Momentum**	10^{-4}	0.919516	0.918453	0.923631	0.919516		
	10^{-5}	0.925039	0.923380	0.926142	0.925039		
	10^{-6}	0.841136	0.827947	0.837552	0.841136		
**None**	-	0.148869	0.046051	0.027321	0.148869		
**FastViT 7 class (Normal, Covid-19, Viral Pneumonia, Bacterial Pneumonia, MERS, SARS, Tuberculosis)**							
	**Learning Rate**	**Accuracy**	**F1-score**	**Precision**	**Recall**		
**Adam**	10^{-4}	0.970016	0.969998	0.971091	0.970016		
	10^{-5}	0.954235	0.953971	0.957553	0.954235		
	10^{-6}	0.814571	0.793420	0.802834	0.814571		
**AdamW**	10^{-4}	0.970542	0.970589	0.972016	0.970542		
	10^{-5}	0.949763	0.949210	0.952920	0.949763		
	10^{-6}	0.832194	0.814165	0.817709	0.832194		
**NAdam**	10^{-4}	0.976328	0.976355	0.977391	0.976328		
	10^{-5}	0.958443	0.958130	0.958766	0.958443		
	10^{-6}	0.823777	0.808304	0.813316	0.823777		

**RAdam**	10^{-4}	0.970805	0.971047	0.972409	0.970805		
	10^{-5}	0.937927	0.936963	0.941823	0.937927		
	10^{-6}	0.803261	0.781623	0.790443	0.803261		
**SGDW**	10^{-4}	0.942925	0.941877	0.943183	0.942925		
	10^{-5}	0.855076	0.840472	0.839329	0.855076		
	10^{-6}	0.580747	0.460949	0.478760	0.580747		
**Momentum**	10^{-4}	0.933719	0.932348	0.934996	0.933719		
	10^{-5}	0.865071	0.852910	0.851486	0.865071		
	10^{-6}	0.612572	0.500666	0.505154	0.612572		
**None**	-	0.061021	0.051851	0.181815	0.061021		
**CrossViT 7 class (Normal, Covid-19, Viral Pneumonia, Bacterial Pneumonia, MERS, SARS, Tuberculosis)**							
	**Learning Rate**	**Accuracy**	**F1-score**	**Precision**	**Recall**		
**Adam**	10^{-4}	0.920831	0.922684	0.934612	0.920831		
	10^{-5}	0.969490	0.969082	0.969999	0.969490		
	10^{-6}	0.900053	0.891989	0.897481	0.900053		
**AdamW**	10^{-4}	0.928459	0.926242	0.927072	0.928459		
	10^{-5}	0.969490	0.969374	0.969824	0.969490		
	10^{-6}	0.905313	0.900745	0.909173	0.905313		
**NAdam**	10^{-4}	0.925302	0.926511	0.943441	0.925302		
	10^{-5}	0.955287	0.955108	0.957735	0.955287		
	10^{-6}	0.902946	0.894019	0.891984	0.902946		
**RAdam**	10^{-4}	0.949237	0.949197	0.949755	0.949237		
	10^{-5}	0.959758	0.958703	0.961348	0.959758		
	10^{-6}	0.884797	0.878160	0.881718	0.884797		
**SGDW**	10^{-4}	0.865597	0.858335	0.865369	0.865597		
	10^{-5}	0.709627	0.637596	0.683058	0.709627		
	10^{-6}	0.536297	0.374425	0.287614	0.536297		
**Momentum**	10^{-4}	0.847712	0.838191	0.839863	0.838191		
	10^{-5}	0.715150	0.643188	0.725742	0.715150		
	10^{-6}	0.536297	0.374489	0.287690	0.536297		
**None**	-	0.227249	0.267090	0.361958	0.227249		

Supplementary Table 4. Estimation of the model performance by calculating F1-scores of each class

The table shows the F1-scores for predicting each class in the data sets of 4 Class Dataset of ViT, 7 Class Dataset of ViT, 7 Class Dataset of FastViT, and 7 Class Dataset of CrossViT using different optimizers of Adam, AdamW, NAdam, RAdam, SGDW, and Momentum, respectively. The models are tested with each learning rate 10^{-4}, 10^{-5}, and 10^{-6}.

**ViT 4 class F1-score each class**								
	**Learning Rate**	**Normal**	**Covid-19**	**Viral Pneumonia**	**Bacterial Pneumonia**			
**Adam**	10^{-4}	0.961373	0.879220	0.549391	0.635762			
	10^{-5}	0.979082	0.941860	0.820097	0.898039			
	10^{-6}	0.959431	0.884903	0.865350	0.952919			
**AdamW**	10^{-4}	0.975481	0.930909	0.552866	0.596244			
	10^{-5}	0.978345	0.943043	0.789560	0.887795			
	10^{-6}	0.964200	0.896296	0.881119	0.947170			
**NAdam**	10^{-4}	0.960555	0.879705	0.524457	0.619154			
	10^{-5}	0.972222	0.916851	0.825336	0.926523			
	10^{-6}	0.962857	0.895323	0.843750	0.936130			
**RAdam**	10^{-4}	0.949755	0.822785	0.592857	0.793265			
	10^{-5}	0.980515	0.944928	0.869565	0.939623			
	10^{-6}	0.968900	0.911095	0.833333	0.911736			
**SGDW**	10^{-4}	0.952470	0.849294	0.711730	0.884342			
	10^{-5}	0.951300	0.856061	0.789762	0.915349			
	10^{-6}	0.901019	0.663844	0.530841	0.781940			
**Momentum**	10^{-4}	0.955723	0.863777	0.675781	0.864528			
	10^{-5}	0.947591	0.833073	0.789223	0.878486			
	10^{-6}	0.908635	0.683761	0.532872	0.771014			
**None**	-	0.466955	0.207567	0.000000	0.000000			
**ViT 7 class F1-score each class**								
	**Learning Rate**	**Normal**	**Covid-19**	**Viral Pneumonia**	**Bacterial Pneumonia**	**MERS**	**SARS**	**Tuberculosis**
**Adam**	10^{-4}	0.980703	0.914761	0.553247	0.633110	0.610169	0.637681	0.954128
	10^{-5}	0.974433	0.928367	0.769748	0.887160	0.892857	0.925926	0.972644
	10^{-6}	0.962013	0.884903	0.892473	0.958724	0.867925	0.943396	0.987654
**AdamW**	10^{-4}	0.969712	0.875652	0.526490	0.739837	0.617647	0.508475	0.823834
	10^{-5}	0.981923	0.945481	0.802610	0.891283	0.846154	0.962963	0.990654
	10^{-6}	0.958956	0.874719	0.912088	0.969529	0.775510	0.836364	0.990712
**NAdam**	10^{-4}	0.969375	0.863870	0.604585	0.710831	0.705882	0.541667	0.902857
	10^{-5}	0.979307	0.939568	0.731118	0.831088	0.888889	0.928571	0.990654
	10^{-6}	0.962928	0.892054	0.910420	0.971323	0.836364	0.909091	0.987654
**RAdam**	10^{-4}	0.975321	0.910500	0.531758	0.671503	0.679245	0.658228	0.885794
	10^{-5}	0.987274	0.967830	0.862129	0.937381	0.862745	0.945455	0.987654
	10^{-6}	0.971936	0.908957	0.938547	0.977252	0.836364	0.884615	0.963636
**SGDW**	10^{-4}	0.965566	0.881432	0.735669	0.850851	0.723404	0.807692	0.975460
	10^{-5}	0.958760	0.882698	0.890943	0.966667	0.734694	0.846154	0.984520
	10^{-6}	0.893435	0.596939	0.542574	0.828625	0.000000	0.000000	0.853933
**Momentum**	10^{-4}	0.962699	0.876304	0.773649	0.883721	0.750000	0.791667	0.960961
	10^{-5}	0.952899	0.853095	0.845173	0.944705	0.723404	0.754717	0.987578
	10^{-6}	0.902245	0.669421	0.712727	0.879621	0.000000	0.071429	0.890966
**None**	-	0.000000	0.000000	0.000000	0.290552	0.029412	0.000000	0.082915
**FastViT 7 class F1-score each class**								
	**Learning Rate**	**Normal**	**Covid-19**	**Viral Pneumonia**	**Bacterial Pneumonia**	**MERS**	**SARS**	**Tuberculosis**
**Adam**	10^{-4}	0.982617	0.957827	0.909747	0.969302	0.912281	0.842105	1.000000
	10^{-5}	0.975985	0.922964	0.894118	0.944762	0.807018	0.830189	0.993789
	10^{-6}	0.889036	0.567351	0.662813	0.864175	0.000000	0.000000	0.850174
**AdamW**	10^{-4}	0.984443	0.954128	0.920415	0.958647	0.928571	1.000000	0.996885
	10^{-5}	0.970813	0.905295	0.910959	0.953817	0.736842	0.745763	0.993789
	10^{-6}	0.908069	0.654362	0.598291	0.854895	0.000000	0.000000	0.847458

**NAdam**	10^{-4}	0.988835	0.969525	0.922261	0.962687	0.931034	0.981818	0.993789
	10^{-5}	0.974879	0.926619	0.915572	0.965580	0.771930	0.779661	0.996885
	10^{-6}	0.905660	0.651085	0.637838	0.820225	0.000000	0.000000	0.807273
**RAdam**	10^{-4}	0.987739	0.963372	0.916376	0.955951	0.800000	0.819672	0.993789
	10^{-5}	0.962681	0.883234	0.873096	0.934866	0.816327	0.925926	0.990712
	10^{-6}	0.882405	0.619247	0.538813	0.850655	0.000000	0.000000	0.674797
**SGDW**	10^{-4}	0.964115	0.887583	0.910420	0.956602	0.754717	0.730769	0.975610
	10^{-5}	0.921005	0.695582	0.670833	0.879433	0.000000	0.000000	0.914286
	10^{-6}	0.727501	0.040921	0.000000	0.432361	0.000000	0.000000	0.000000
**Momentum**	10^{-4}	0.957386	0.879349	0.862963	0.954717	0.678571	0.711111	0.975610
	10^{-5}	0.920102	0.731250	0.744681	0.885329	0.000000	0.000000	0.915361
	10^{-6}	0.747204	0.040712	0.007246	0.629162	0.000000	0.000000	0.000000
**None**	-	0.014299	0.130969	0.070951	0.061538	0.011806	0.032258	0.117302
**CrossViT 7 class F1-score each class**								
	**Learning Rate**	**Normal**	**Covid-19**	**Viral Pneumonia**	**Bacterial Pneumonia**	**MERS**	**SARS**	**Tuberculosis**
**Adam**	10^{-4}	0.985936	0.957404	0.653903	0.764092	0.872727	0.923077	0.969512
	10^{-5}	0.979011	0.934783	0.959854	0.979964	0.888889	0.947368	0.993789
	10^{-6}	0.945889	0.797574	0.822934	0.911368	0.000000	0.071429	0.981481
**AdamW**	10^{-4}	0.970930	0.919605	0.683438	0.899734	0.840000	0.901961	0.906516
	10^{-5}	0.982327	0.952837	0.923650	0.971793	0.816327	0.896552	0.987654
	10^{-6}	0.947493	0.831111	0.809524	0.907336	0.550000	0.137931	0.942943
**NAdam**	10^{-4}	0.984712	0.946771	0.709040	0.785791	0.846154	0.941176	0.958084
	10^{-5}	0.979454	0.941176	0.863946	0.933078	0.708333	0.867925	0.996885
	10^{-6}	0.948676	0.809125	0.823932	0.913251	0.000000	0.000000	0.945783
**RAdam**	10^{-4}	0.982917	0.948649	0.795455	0.909910	0.851852	0.892857	0.943620
	10^{-5}	0.970623	0.905830	0.962825	0.990009	0.816327	0.915254	0.963855
	10^{-6}	0.943831	0.798502	0.730964	0.864341	0.342857	0.000000	0.942249
**SGDW**	10^{-4}	0.943131	0.794603	0.635332	0.788900	0.129032	0.000000	0.958466
	10^{-5}	0.815843	0.405512	0.064516	0.811715	0.000000	0.000000	0.000000
	10^{-6}	0.698168	0.000000	0.000000	0.000000	0.000000	0.000000	0.000000
**Momentum**	10^{-4}	0.941369	0.753349	0.587248	0.778744	0.000000	0.000000	0.828338
	10^{-5}	0.828076	0.399623	0.007407	0.819620	0.000000	0.000000	0.072289
	10^{-6}	0.698288	0.000000	0.000000	0.000000	0.000000	0.000000	0.000000
**None**	-	0.411525	0.002736	0.040000	0.294780	0.006441	0.005277	0.000000
